# How does explicit knowledge inform policy shaping? The case of Burkina Faso’s national social protection policy

**DOI:** 10.1371/journal.pone.0284950

**Published:** 2023-04-27

**Authors:** Kadidiatou Kadio, Christian Dagenais, Valery Ridde

**Affiliations:** 1 Institut de Recherche en Sciences de la Sante (IRSS) du Centre National de la Recherche Scientifique et Technologique (CNRST) Siège à Wemtenga, Ouagadougou, Burkina Faso; 2 Fellow Pilote African Postdoctrorat Academy - PAPA, Goethe University Frankfurt, Frankfurt, Germany; 3 Department of Psychology, University of Montreal, Pavillon Marie-Victorin, Montréal, Canada; 4 French Institute for Research on Sustainable Development (IRD), CEPED (IRD-Université Paris Descartes), Université Paris Sorbonne Cités, ERL INSERM SAGESUD, Paris, France; 5 Institut de Santé et Développement, Université Cheikh Anta Diop, Dakar, Sénégal; University of Sargodha, PAKISTAN

## Abstract

In 2009, Burkina Faso embarked on a process leading to the development of a national social protection policy (politique nationale de protection sociale–PNPS) in 2012. The objective of this study was to analyze the circumstances under which explicit knowledge was used to inform the process of emergence and formulation PNPS. The term explicit knowledge excludes tacit and experiential knowledge, taking into account research data, grey literature, and monitoring data. Court and Young’s conceptual framework was adapted by integrating concepts from political science, such as Kingdon’s Multiple Streams framework. Discursive and documentary data were collected from 30 respondents from national and international institutions. Thematic analysis guided the data processing. Results showed that use of peer-reviewed academic research was not explicitly mentioned by respondents, in contrast to other types of knowledge, such as national statistical data, reports on government program evaluations, and reports on studies by international institutions and NGOs, also called technical and financial partners (TFPs). The emergence phase was more informed by grey literature and monitoring data. In this phase, national actors deepened and increased their knowledge (conceptual use) on the importance and challenges of social protection. The role of explicit knowledge in the formulation phase was nuanced. The actors’ thinking was little guided by the question of whether the solutions had the capacity to solve the problem in the Burkina Faso context. Choices were based very little on analysis of strategies (effectiveness, equity, unintended effects) and their applicability (cost, acceptability, feasibility). This way of working was due in part to actors’ limited knowledge on social protection and the lack of government guidance on strategic choices. Strategic use was clearly identified. It involved citing knowledge (reports on studies conducted by TFPs) to justify the utility and feasibility of a PNPS. Instrumental use consisted of drawing from workshop presentations and study reports when writing sections of the PNPS. The consideration of a recommendation based on explicit knowledge was influenced by perceived political gains, i.e., potential social and political consequences.

## Introduction

Social protection, long considered costly, has regained prominence in the policies of low- and middle-income countries [1, 2]. The concept of social protection is used interchangeably with several other concepts to designate or describe various interventions, such as mandatory provisions to provide security to citizens faced with the risks of old age, disability, or unemployment (ISSA, 2015), or regular social transfers to poor or vulnerable populations to support their consumption and their accessibility to certain basic services (education, health) [3].

Social protection policies and programs are aimed at reducing poverty and vulnerability to unemployment, social exclusion, illness, disability, and aging by helping people to cope with these risks when they occur [1]. They are both contributory (social insurance) and non-contributory (conditional or unconditional social transfers) in nature, with the ultimate aim of reducing the risks and socio-economic vulnerability that are understood to be the main causes of poverty [4, 5].

Since the launch of the United Nations Social Protection Floor initiative in 2009 [1], the African Union has recommended that the right to social protection be incorporated into national legislation and constitutions [6], countries have formulated social protection programs as part of national development plans [7, 8]. The initial focus has been on social safety net programs, including cash and non-cash transfers and exemptions from payment for health and school services [9, 10]. However, some countries have opted for a systemic approach through the development of national social protection strategies or policies. These policies define a long-term national vision, social protection priorities, objectives to be achieved, and implementation strategies [11]. They support the idea that social protection, as a social guarantee provided by the State, makes the State accountable to citizens, strengthens the social contract between the two, and legitimizes pro-poor policy priorities [12].

Most research on social protection has been funded and commissioned by donors and, as such, carries the risk of promoting a particular vision, is focused on social safety net programs, and deals mostly with their implementation and effectiveness, sometimes raising debates on formulation but without in-depth studies [13–16]. Indeed, the research deals primarily with technical aspects, such as measuring the impact of social transfer programs or systems for targeting beneficiaries (how to target accurately and cost-effectively). This research has shown that transnational actors have been very influential in social policy reform in much of Africa [17, 18]. All major international agencies involved in development have adopted a commitment to social protection that is explicitly included in sustainable development objectives. These donors and international partners have been urging governments to put in place social protection programs [7, 8].

An empirical study of 12 cases showed that the emergence of social transfer programs is the result of complex interactions between groups of international and national actors with different views on social protection and financial responsibility [9]. In some regions of Africa (Central, East, and West), bilateral and multilateral organizations and international NGOs have exerted a significant influence on the emergence of social transfers [18–20]. Ghana is one of the first West African countries to adopt a social transfer program, the Livelihood Empowerment against Poverty LEAP) program, under strong donor influence [21].

Although the majority of social transfers are designed by international organizations, governments sometimes decide on the content against the advice of these organizations [22, 23]. They have often rejected social transfer reforms approved and supported by donors [24]. In Zambia and Tanzania, governments have long resisted pressure from international actors to invest domestic funds in unconditional cash transfers and to integrate them into comprehensive social protection strategies [25, 26].

The social transfer policy development process has also been examined from the perspective of the dissemination of learning and policy innovations [20, 27]. This approach is often descriptive and shows how diffusion entrepreneurs at the international level rely on different types of knowledge and strategies to disseminate policies [28]. In contrast, little attention has been paid to local processes for the appropriation of innovations [29] and of locally produced explicit knowledge (see next section regarding definition). While the analysis of scientific knowledge use clearly takes into account the role of local context and the attitudes of national actors (decision-makers and producers of scientific knowledge), the place of local knowledge is rarely addressed [30–33]. These findings underscore the need for knowledge about the processes by which national actors use explicit knowledge to guide their thinking. It is important to know more about how national actors orient their decision-making. Specifically, the present study draws on the case of the PNPS in Burkina Faso to understand the processes involved.

In 2009, Burkina Faso embarked on a process that led to the development of a national social protection policy (*politique nationale de protection sociale*–PNPS) in 2012. The objective of this study was to analyze how explicit knowledge was used to inform the two phases of the PNPS development process, i.e., emergence and formulation. It complements previous studies on the social protection policy-making process in Burkina Faso [34–37] by focusing a process analysis on the use of explicit knowledge to guide choices during the development of social protection policies. This study was aimed at determining: 1) what types of explicit knowledge were consulted and how this knowledge was used to guide their choice; and 2) what were the issues shaping use, what factors that can influence the use of explicit knowledge in decision-making.

### Analytical framework

We drew on the RAPID framework [38] and the field of public policy studies[39–41]. From this policy perspective, we analyzed the influence of context and of actors, as well as how the characteristics of explicit knowledge influenced its course in policy development[38, 42, 43].

#### The use of explicit knowledge in a complex decision-making context

The context in which a policy is developed is comprised of all the political, economic, ideological, and normative factors that can influence the use of explicit knowledge in decision-making[38, 44]. These factors help create the conditions leading to knowledge use, such as positive opportunities and a more receptive attitude in the decision-making environment [40, 45, 46]. The context in which public policy is developed is highly political, rapidly changing, and depends on a variety of factors, inputs, and relationships [47]. Beyond explicit knowledge, decision-makers make judgments about institutional interests based on what is most advisable given the context and circumstances [48]. Choices are influenced by available resources, bureaucratic culture, pressure groups, ideas, values, and the need for an immediate response to the events [49–51]. Policy development is a complex process resulting a construct infused by the actors’ experiences and values [52]. In this decision-making context, described as a complex social dynamic [53], knowledge use becomes difficult to identify and categorize [50, 54]. To understand the flow of knowledge, this complexity must be taken into account and policy-making must be seen as more than a one-time act attributable to clearly identified, authoritative “decision-makers” [42, 55, 56].

#### Explicit knowledge

**Fig 1:**
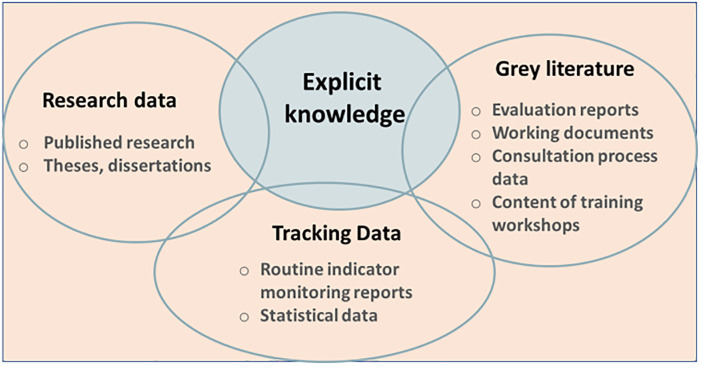
What we mean by explicit knowledge.

What is considered knowledge in policy-making is subject to debate. A systematic review of the literature [57] revealed widespread use of informal knowledge (local data or tacit knowledge). Considering only peer-reviewed research data yields a narrow definition of knowledge, attributed to the rationalist epistemological position of evidence-based medicine [31]. As well, information from academic research data is less used than is information coming from expert opinions, personal experience, internal documents or legal regulations, and government reports of community complaints and opinions [33, 58]. In light of these considerations, Oliver, Innvar (57) argue that any analysis of knowledge use must take into account knowledge beyond the confines of research findings. The present study opted for this broad view by adopting the term “explicit knowledge” [59] to take into account unpublished research and evaluation reports, routine indicator monitoring reports, results of consultation processes, population surveys, and statistical data (Fig 1). Explicit knowledge about a phenomenon can modify the stances taken by the actors, who will then perceive it as requiring public action [40, 60]. Technical information can be used to frame a public problem by helping actors understand the issues, and then to shape policy directions by contributing to the development of ideas and social movements [61]. Knowledge about a solution’s costs, effectiveness, or acceptability can inform choices during policy development [40, 62]. If the supply (availability, access, clarity, quality, relevance, reliability of research results, costs) of knowledge is inadequate to satisfy the demand, this will influence its credibility and acceptability and may limit its use [56, 57]. A few studies have shown that locally produced knowledge, specifically that derived from scientific research, has little influence on social health protection policies [63]. Contextual factors, such as financial constraints, lack of confidence in local research, and political issues (politics) negatively influence the use of local research in health policy making in Africa [63, 64].

#### The actors

Actors are the channels through which knowledge passes to influence decision-making. These are individuals or institutions either affected by the change or who have a stake in the effects of the policy even if they derive no direct benefit themselves [38]. The active participants in policy development are governmental and non-governmental actors representing personal or collective interests [65–67]. Often involved in networks or communities of practice, they can play different roles [68, 69]. Grouped into coalitions, they use knowledge to nurture and strengthen their deep beliefs, convictions, and values, with a view to consolidating their approaches to argue the influence of a political decision [41]. Political entrepreneurs can use knowledge related to their ideas and values to draw the attention of decision-makers to a situation so that it becomes a public problem [40]. Empirical studies show that international organizations and financial institutions have influenced the introduction and design of social transfer programs through knowledge-sharing strategies: technical assistance to ministries, and the participation of political leaders and senior officials in conferences, training seminars, and study trips [24, 70–72]. They have also sponsored and shared research on benefits, costs, and policy design [8, 24].

#### Types of knowledge use

Research-based knowledge is a source of information to improve policies when used in decision-making [73, 74]. This well-known benefit has reinforced the idea that social protection policies should build on international successes while at the same time being rooted in the specific context of each country [1]. Consensus has been reached on three types of scientific knowledge use. Instrumental use involves the direct use of knowledge to solve problems [75, 76]. Conceptual use occurs when the decision-maker acquires new knowledge about a situation or phenomenon. Symbolic or strategic use refers to using knowledge to support the continuance of an established position or to justify a decision [76]. Research evidence that has shown that the positive impact of cash transfer programs has helped legitimize them and support their adoption in Ghana [24]. Publications by DFID, the World Bank, and other international organizations have provided essential knowledge on cash transfer programs and the Latin American experience [24]. This explicit knowledge has supported the argument that cash transfer programs contribute to economic growth [77, 78]. Also, concerns about financial sustainability, the risk of compromising productivity and economic growth, the purported dependence and laziness of the poor, or the idea of a “culture of poverty” and the disempowerment of populations are often invoked to justify the preference of governments for conditional transfer programs, social insurance, subsidies for peasant production and fertilizers, provision of free seeds, and indigent selection through income measurement methods [8, 79–81].

## Methods

### Data collection and analysis

This article presents a case study [82]. Exploratory interviews were conducted with two members of the PNPS Implementation Secretariat to obtain preliminary information on the process, collect documents, and identify key informants. Based on analysis of these initial data, the stages of policy development were chronologically reconstructed and an initial list of respondents was prepared, to which names were added during the informal and semi-structured interviews using the snowball recruitment technique [83]. We asked each respondent to provide names of others who had participated in the processes, and so on, to add to the list of respondents. In total, 55 documents were collected and 42 interviews conducted (10 informal/exploratory and 32 semi-structured and recorded) with 37 respondents (Table 1). Particular attention was given to reserving the anonymity of those interviewed. Participants were asked for free and informed consent after being informed of the pros and cons of participating in the research. Written consent was obtained through an information and consent form signed by each research participant. The information collected will remain confidential.

**Table 1:** Numbers of documents analyzed and of participants interviewed.

Documents analyzed	N
**Government strategy documents** *Accelerated growth and sustainable development strategy* *Prospective Burkina Faso 2025*: *Vision and strategies for the future*	2
**Routine/statistical data**	2
**Grey literature–government** *Evaluation report* *Implementation report* *Minutes of meetings*	11
**Grey literature–international institutions and NGOs** *Study report* *Working document*	9
**Training workshops** *Terms of reference*, *presentation* *Reports*, *concluding notes*	20
**PNPS process** *Framework document* *Reports from specialized commissions* *Steering committee report* *PNPS document*	11
***TOTAL documents***	**55**
**Interviews**	
**Informal** *Government actors* *International institutions and NGOs*	10
**Semi-structured**	
***Government actors (managers and senior officials)*** *Member*, *inter-ministry PNPS development committee* *Director*, *Poverty Reduction Strategic Framework (CSLP) Office* *Permanent PNPS Implementation Secretariat* *Permanent Health Insurance Secretariat*	20
***Local civil society organizations*** *Permanent Secretariat*, *NGO Coordination (SPONG)* *ASMAD (Association Songuimanégré / Aide au développement endogène)* *RAMS (Réseau d’Appui aux Mutuelles de Santé)*	3
***International institutions / international NGOs*** *UNICEF*, *World Bank*, *World Food Programme* *HELP*, *Helvetas*, *OXFAM*, *ECHO*, *Terre des Hommes*	9
***TOTAL interviews***	**42**

The data were collected between November 2015 and July 2016. Approval was obtained from Burkina Faso’s health ethics committee and the health research ethics committee of the University of Montreal (CERAS-2015-16-178-D).

We have identified difficulties inherent in the retrospective approach to process analysis, likely to negatively influence the quality of our research. First, the memory bias linked to the difficulty of remembering past events. Then, the memory bias due to staff mobility. We have nevertheless tried to reduce this bias through the diversity of data collection sources and the triangulation of information.

Thematic discourse analysis was used to process the data [84]. The analysis was both inductive and deductive. The interviews were fully transcribed and then coded using NVivo 11 software. From a first, inductive coding, performed without an analysis grid, a number of themes emerged. A second, more deductive analysis was performed based on the analytical framework to better describe and explain the themes and sub-themes observed inductively. In parallel with the thematic analysis of interviews, the documents were organized chronologically to construct a historical account of the events related to the policy’s development phases (emergence and formulation) from 2008 to 2012. This made it possible to monitor context, links (actors), and knowledge in tandem. Reports of key events were reviewed to identify the opportune moments, the actors, and their strategies, and then to analyze their contribution in bringing knowledge into the decision-making sphere. Triangulation of the respondents’ statements and the document contents, bearing in mind two questions (what information was useful for the emergence or the formulation, and how the information was used), highlighted the types of knowledge and the actors’ roles and strategies.

## Results

The PNPS is divided into six programs aimed at: (1) improving social benefits for the poor and vulnerable; (2) improving access to basic social services for the poor and vulnerable; (3) safeguarding employment and guaranteeing a minimum income for the poor; (4) expanding social benefits coverage for workers in both the formal and informal sectors; (5) improving governance of the PNPS; and (6) strengthening the capacity of actors in the system [34].

### The context of PNPS development and access to explicit knowledge

Policy development includes both the emergence, or agenda setting, phase and the formulation phase. Agenda setting concerns the way in which certain problematic situations manage to compel the attention and intervention of public authorities [68]. The global context of the promotion of a social protection floor strengthened the receptiveness of the Burkina Faso government to the development of a PNPS in response to the social protection deficit in the country. The international institutions, under the impetus of UNICEF, had come together in a network of political entrepreneurs to promote this solution, in line with their mandate, and to convey ideas on social protection that coincided with the government’s current expectations and needs (national solidarity, peace, and social cohesion).

The formulation stage consisted of developing solutions that could be applied to the situations to be regulated [68]. In February 2010, on instructions of the Prime Minister, the Minister of the Economy and Finance (MEF) set up an inter-ministerial institutional mechanism bringing together members of the public administration and representatives of technical and financial partners (TFPs), non-governmental organizations (NGOs), and local associations. This committee was responsible for leading the drafting of policy and coordinating the work of two commissions, on social insurance and on social safety nets. The policy was formulated between February 2010 and September 2012, following a process organized in three periods. During the first eight months, the institutional coordination mechanism was put in place; the terms of reference and frameworks were drawn up. The second period of 17 months was devoted to developing the PNPS, which included drafting the reports of the two commissions as well as the policy. During the last three months of the process, two national validation workshops were held and the policy was adopted by the Council of Ministers.

International actors/agencies contributed significantly to improving local actors’ knowledge of social protection through several strategies, with the aim of influencing the PNPS development process.

#### Producing knowledge to support advocacy and policy dialogue

To begin, TFPs conducted studies to improve their own understanding of the situation, in order to better influence decisions.

In 2008, a World Food Programme (WFP) study on the food crisis in Burkina Faso provided an overview of the people affected and was used to plan a food stamp distribution. The evaluation results from this intervention were used to support discussions with the government aimed at developing a national program. Similarly, a study on social safety nets was primarily intended to provide the World Bank with contextual knowledge for the planning and implementation of its project.

UNICEF commissioned studies to better understand Burkina Faso’s specific situation with respect to social protection. A first study of the impact of the economic crisis on social protection showed the poor were more affected [85]. In response to the MEF’s questions on the government’s ability to finance social safety net programs, UNICEF commissioned a second study on tax revenues and public spending. The study showed that cash transfers were economically sustainable by the government and had a potential positive impact on poverty reduction [86]. The results were used in the dialogues to show the MEF that implementing cash transfers would have no negative impact on the economy.

#### Results reporting sessions and government meetings as channels for disseminating local knowledge

In addition to producing knowledge on the national context, TFPs used or created opportune moments to disseminate it. They organized meetings with key actors to disseminate the main findings of their research. These conversations raised the need for deep reflection on social protection that would look beyond social safety net programs.

“*They presented the study’s findings*. *They said*: *listen*, *social safety nets are only one component of social protection*, *particularly the non-contributive component*. *There are other contributive components*, *like social insurance*, *health insurance*. *So this required some reflection*”

[AM-M2].

They also used existing government sharing and consultation frameworks to disseminate knowledge. The studies’ results and preliminary recommendations were presented at the annual review of the Poverty Reduction Strategic Framework (*Cadre stratégique de lutte contre la pauvreté*—CSLP) chaired by the Prime Minister in April 2010. Discussions focused on shortcomings and challenges, and on the studies’ conclusions and recommendations.

#### Study and training travel

The TFPs funded study and training travel for their Burkinabè collaborators. These trips were intended to provide access to knowledge that would help change their perceptions and vision of social protection. For example, the concept of the social safety net was incorporated into the vocabulary of the Ministry of Social Action (MAS) in 2009 following a training workshop in Senegal.

“*The term social safety net was unknown before 2009*. *It was the Ministry’s SG [general secretariat] who were the first to go to Dakar for training on social safety nets and social protection in 2009 and who brought this term back to the Ministry*”

[AS-M2].

Likewise, the African Platform for Social Protection funded a trip in 2011, which enabled the Permanent Secretariat for NGO Coordination (SPONG) to obtain information on the experiences of other countries in the network. This study tour partly generated momentum at SPONG, which then took steps to become involved in the PNPS development process that had been underway since 2010 [34].

#### National training workshops

Two training workshops were organized in January and April 2010 by the MEF in collaboration with the TFPs. The first brought together a dozen ministries and TFPs involved in the field of social protection. The second was attended by the same actors as well as high-level decision-makers.

The objective of these workshops was to improve participants’ knowledge about social protection. These workshops used explicit knowledge strategically to argue for intersectoral dialogue, consensus on the need for a comprehensive policy, and a decision to develop a national strategy for social safety nets. One TFP representative explained:

“*Here’s a typical example; I’m completely guilty of this*. *If we say something is evidence-based*, *but we only take what fits with our opinion*. *And most often*, *that’s it*. *Because there was so much reluctance about transfers*, *people weren’t convinced*, *so everything became advocacy*. *And likewise*, *the workshop was an advocacy session*. *It had been focused on cash transfers*, *and gradually got the idea across that cash transfers are a good thing*, *that’s how it usually went*, *it was to show it was possible*”

[UNC-F1].

The TFPs fitted the knowledge to their own objectives (ignoring contradictory knowledge) to support their ideas and opinions and to have these accepted. A PTF “asserts:*Frankly*, *it was advocacy*, *it wasn’t to say uh*, *let’s look at the evidence and then*, *we wouldn’t have talked about a project that didn’t work*. *We said the things not to do*, *but the idea was to move towards the agenda of money transfer*, *monetary transfers*.*” A-P1*

One session was dedicated to experiences of social projects and programs and to social protection initiatives. It was aimed at justifying the need for a government decision targeting poor and vulnerable people. The reports of the workshops indicate that they enabled ministries to share information and to dialogue on the main challenges and opportunities related to social protection. Based on a synthesis of the studies, a comprehensive review of social protection and a roadmap for developing the PNPS were drafted.

In short, the TFPs relied on explicit knowledge produced in Burkina Faso and elsewhere to help government actors understand the importance of investing in social protection, as well as to see the opportunities and the need for a PNPS. The workshops and meetings served as a knowledge dissemination channel. These interactive encounters fostered discussion and a better understanding of social protection, provided an overview of experiences elsewhere, and led to a consensus on the need for a comprehensive policy response to the social protection shortfall.

### Types of explicit knowledge and of use to inform the PNPS development

This section presents the types of knowledge used by actors in the emergence and formulation phases. Three types of explicit knowledge informed the PNPS formulation process: routine data from the ministries (daily tracking data or statistical data), data from government grey literature (project and program implementation reports, evaluation reports), and data from the grey literature of TFPs and NGOs: working documents, evaluation reports, orientation documents, and training workshop presentations. Scientific research published in peer-reviewed journals was not explicitly mentioned.

Viewing policy development as the result of a process influenced by many factors, including explicit knowledge, we were unable to identify accurately any instrumental use of explicit knowledge in the emergence of the PNPS. However, the analysis of this phase of the PNPS development process showed that contextual and situational factors (drop in purchasing power, social tension, food insecurity) had created a need for knowledge among government actors and TFPs to better understand the situation. Explicit knowledge from reports of studies commissioned by TFPs, evaluation reports on the implementation of projects, programs, and policies (governmental and non-governmental), as well as routine monitoring data and statistical data enlightened national actors on the need for change, with a view to improving social protection policies. Conceptual use of this knowledge helped shape stakeholders’ perceptions and understanding by informing them of the various contours of social protection: the social protection deficit (extent, persons concerned, consequences), the causes of that deficit (response not adapted to needs, situation not new), the values and expectations of populations, the link between social protection and poverty (the consequences of the economic crisis on vulnerable people). This conceptual use of the knowledge produced led to an understanding that the social protection deficit was a consequence of low state investment in the social sector and the inefficiency of the few actions implemented. Furthermore, poor and vulnerable people were perceived as being more affected by the social protection deficit, hence the decision to formulate a PNPS.

The use of explicit knowledge to inform the PNPS formulation was nuanced. Activity reports of the ministries, capitalization documents of the pilot projects, documents and reports on social protection from the TFPs (UNICEF, World Bank, ILO), and basic documents and presentations of the social protection training workshops were all used to clarify concepts and identify PNPS strategies. This mapping could be seen as instrumental use of explicit knowledge to write the content of the PNPS, since the policy strategies were drawn from the content of the reports. However, some actors (TFPs and national actors) considered that the PNPS did not represent a choice informed by these strategies’ capacity to solve the social protection deficit problem (knowledge on the effectiveness of the strategies). They described the PNPS as an aggregation of all that was possible in terms of social protection, and ultimately as the result of planning based on the ministries’ institutional missions, rather than as a choice informed by assessing the strategies’ capacity to achieve the objectives set (reducing the social protection deficit). For others (TFPs and national actors), the strategies selected were those that had proven their effectiveness through pilot projects in Burkina Faso or elsewhere. However, there was a clear strategic use that consisted of drawing on documents (study reports, evaluations, statistics) to justify the PNPS content: setting out the foundations and guiding principles of the PNPS, justifying the need to formulate a policy, illustrating the extent of the social protection deficit and the ineffectiveness of the CSLP, and supporting the feasibility and relevance of a specific strategy.

### Knowledge and type of use to explain the development of a PNPS

This section shows how explicit knowledge was used to put social protection on the government agenda and then to inform the decision to formulate a PNPS, as well as to choose the content of the PNPS.

Table 2 summarizes the type of knowledge used in each phase of the PNPS development process, with illustrations by type of use.

**Table 2:** Types of knowledge and of use according to the PNPS development process phases.

Type / source of knowledge	Type of use in PNPS development	
	Emergence	Formulation
**Routine data** Monitoring indicator Early alert system	**Conceptual use** Grasping the magnitude of the situation	
**Grey literature / government** Evaluation report Statistical yearbook, national survey, poverty profile Prospective study	**Conceptual use** Acquiring knowledge about the problem (magnitude, people involved, consequences) Acquiring knowledge about the causes of the problem (poor fit between response and need, long-standing situation) Acquiring knowledge on populations’ values and expectations	**Strategic use (cited in the PNPS)** Using statistical data from national surveys to illustrate situations (rise in poverty) **Strategic use (cited in the PNPS Framework memorandum)** Explaining the premises and guiding principles of PNPS Illustrating the need to develop a policy Illustrating the magnitude of the social protection shortfall Illustrating the ineffectiveness of Poverty Reduction Strategic Framework (CSLP)
**Grey literature / reports from TFP/NGO studies** Pilot project evaluation report Reports on studies Working document	**Conceptual use** Understanding the impacts of the economic crisis on vulnerable persons Understanding the social protection shortfall Understanding the link between social protection and poverty **Strategic use (basic social protection document)** Citing a study on Malawi to present cash transfers as a solution to the social protection shortfall and illustrate their feasibility in Burkina Faso Supporting the idea of school canteen programs as an opportunity to strengthen social protection and education: citing a study on the positive effect of school canteens on girls’ schooling	**Conceptual use** Using the ILO’s Blanchet report to better understand the underlying concept of SP. Reading the World Bank report on social safety nets, as well as training workshop presentations, to support their understanding of concepts (social insurance, social safety nets, etc.) **Instrumental use** Drawing from the content of training workshop presentations to draft the framework memorandum. Using the content of the World Bank report on social safety nets as a basis for writing the PNPS section on the status of interventions.

#### Knowledge and the decision to develop a PNPS: Emergence of a public problem

Conceptual use was more apparent during the emergence process. With this knowledge, the actors came to understand that the government was being accused of doing nothing, especially for the poor, who are without social protection. They learned about the social protection deficit, the expectations of the population, and the challenge of social protection. All of this explicit knowledge was used conceptually to begin to comprehend the need to develop a PNPS adapted to population needs.

*Explicit knowledge to raise awareness of the social protection deficit*, *its magnitude*, *its causes*, *and the persons affected*. Respondents indicated that the implementation and evaluation reports from the two Poverty Reduction Strategic Framework (CSLPs) review sessions conducted between 2000 and 2010 identified deficiencies that had contributed to the social protection deficit.

The framework memorandum, a basic document to justify the necessity of developing a PNPS, stated that the strategies for implementing the social component of the CSLPs were not adapted to the situations of the various populations [87, 88]. The very first basic document on PNPS prepared by the MEF in collaboration with UNICEF cited statistical data from the 2009–2010 comprehensive survey on household living conditions to illustrate the magnitude of the situation. Poverty profile studies carried out by the National Institute of Statistics and Demographics (INSD) were used to better define poverty, to perceive its magnitude, and to identify the people concerned. An MEF official explained: “*INSD studies on the dimensions of poverty*, *on vulnerability*, *all this was done*. *That’s what showed a little more clearly the magnitude of the phenomenon and the need to develop and implement a social protection policy*” [EF-1F].

Demonstrations and strikes in 2008 spurred initiatives to find information and solutions. Contextual and economic factors (reduced purchasing power, social tension, food insecurity) led to the search for solutions adapted to the needs of the people affected, as well as to the aspirations of the population at large. An inter-ministry economic monitoring committee was set up to monitor the monthly evolution of certain indicators (commodity prices, employment, etc.) and regularly inform the Prime Minister. Routine data from the Ministry of Agriculture’s early alert system had provided the government with information on the magnitude of the 2008 food crisis. To resolve the crisis, the WFP carried out a situational analysis that enabled the government to identify the people most affected by food insecurity. The analysis recommended implementing social protection measures to strengthen people’s resilience.

*Explicit knowledge to understand Burkinabès’ values and expectations*. The national “Burkina 2025” prospective study presented the vision of the desired future for Burkina Faso. That vision was based on stakeholders’ concerns, representations, and definitions collected through a broad national consultation process. For national solidarity to be effective, the report said, it was necessary: “*to reactivate*, *make explicit and*, *above all*, *respect this duty of solidarity…*. *Solidarity means no Burkinabè is excluded and/or marginalized for any reason*” (p. 110) [89]. From this study, the government understood that the population’s deepest expectations were for solidarity and for social protection to be the underlying principle for development. One respondent who had participated in both the study and the presentation of recommendations, as well as in the PNPS process, explained: “*When we undertook the Burkina 2025 prospective study*, *it became clear that*, *if we didn’t resolve the social problem*, *the issue of social protection could never be developed in Burkina*” [EF-AF].

*Explicit knowledge to grasp the challenges to be addressed*. In 2009–2010, the World Bank and UNICEF, in collaboration with the University of Ouagadougou, conducted a study on social safety nets. Even though the need to resolve the issue of the social protection deficit had been recognized well before this study, it was clear from respondents’ statements that it provided an overall view of the situation. It presented a more structured overview of the social safety nets, their dysfunctions and deficiencies, and made recommendations to implement a balanced system of social safety nets. The study provided national actors with more comprehensive knowledge so they could better understand the problem. It also gave TFPs better knowledge regarding the situation and challenges related to social protection in Burkina Faso. According to one TFP respondent: “*It showed us that several social protection programs existed*, *but that they were poorly organized and lacked coordination”* [UNE-P1].

Several sectoral policies that included social protection objectives were implemented as part of the CSLPs. Despite this, the 2008–2009 economic crisis showed the population was very vulnerable. Explicit knowledge produced on these CSLPs, as well as on the government’s responses to the socio-economic situation in 2008 (study report, implementation report, routine monitoring data, statistical data) made national actors aware of the need for reflection not only on the types of interventions, but also on the identification of beneficiaries of social protection policies. This knowledge also helped them understand that the social protection deficit was a consequence of the State’s low investment in the social sector and of the inefficient implementation of the few previous actions, as well as that the population wanted more national solidarity. As such, the social protection deficit was a public problem that the government determined to resolve by developing a national social protection policy that would strengthen national solidarity.

#### Explicit knowledge and development of the PNPS

Three types of use were noted in the formulation phase: 1) conceptual use, to better ensure understanding of certain concepts of social protection; 2) instrumental use, which consisted in drawing inspiration from the contents of evaluation reports, TFP working documents, and presentations at training workshops, defining priority actions, and drafting the contents of the document; and 3) strategic use, which often consisted in quoting a report document to support a position.

*Drafting of the framework memorandum and specialized commission reports*. A framework memorandum was drafted in October 2010 by the MEF’s CSLP Office, supported by UNICEF. It set out the guidelines for developing the PNPS. National actors acknowledged that they had limited knowledge about social protection at the time of writing and said they relied on existing documents. One member of the drafting team explained: “*We weren’t experts on the matter*, *so we based ourselves on what the specialists had done and then tried to create the necessary framework*” [EF-6M]. In fact, the core drafting committee drew upon two training workshops on social protection, UNICEF’s expertise, and the World Bank study on social safety nets: “*We had the presentations [training workshops] that helped us frame it*. *We had documents from UNICEF*, *and used the World Bank interim report on social safety nets*” [EF-DM]. Documentary analysis showed that the memorandum’s content had been inspired by the training workshops.

The specialized commissions prepared their reports between February and June 2011 under a mandate received from the Executive Secretariat for the PNPS Drafting, a temporary secretariat reporting to the MEF. Each commission report was the product of a survey of actions that had been implemented, based on activity reports prepared by member departments. The commissions also drew inspiration from the TFPs’ approach to organize the content of the reports and to add conceptual clarifications. The head of the Social Insurance Commission explained that they consulted the International Labour Office’s (ILO) Bachelet report on the foundations of social protection. They also consulted the capitalization documents for the pilot projects on healthcare fees exemption implemented by NGOs. One member of the Social Insurance Commission explained that they considered: “*some pilot experiments*, *such as the healthcare fees exemption*, *to see how implementing the policy could promote this social insurance component*” [SS-F2].

*Drafting the PNPS content*. the Executive Secretariat for the PNPS Drafting relied primarily on the framework memorandum, the report of the study on social safety nets, and two commission reports while writing the draft policy from August 2011 to June 2012. The World Bank study, extensively mentioned, inspired the presentation of the section on the status of social protection by category of instrument: social insurance, social safety nets, social assistance services, and legislation. The policy’s vision was based on the Burkina 2025 prospective study: “*Burkina Faso*, *a solidary nation that has a system equipped with sufficient and sustainable mechanisms to protect populations against risks and crises*” (p. 39) [89]. The PNPS programs were focused on the poor and vulnerable. These people, according to respondents, were more affected by the social protection deficit and needed more protection, according to the INSD’s surveys on household living conditions.

However, the role of explicit knowledge was not straightforward. Some respondents described the PNPS strategic directions and programs as resulting from a simple planning process based on the ministries’ policy options, with no knowledge having been used to inform choices. One member of the Executive Secretariat for the PNPS Drafting explained: “*We didn’t refer to any research or studies while we were planning*. *There was no strategic reflection on how to organize actions*, *etc*. *No*, *it was only programming*” [AS-F3]. Others, however, reported that the programs were formulated based on interventions that had produced positive outcomes. Existing strategies were renewed in the policy because they had proven their effectiveness. Some social safety nets, such as school canteens, were included as strategies to support education because of their perceived effectiveness. National and international actors considered that the strategy of healthcare fees exemptions for children under five years was retained because its effectiveness had been proven by research results: “*There were specific studies taken into account*, *studies on healthcare subsidies that had shown the possibility of fully covering the care for pregnant women and children under five years*” [EF-2M].

The PNPS is Burkina Faso’s first social protection policy. In sum, the actors involved in the formulation made conceptual, instrumental, and strategic use of the PNPS: first, to improve their own understanding of social protection, and second, to organize and draft the documents. The priority axes and intervention strategies are taken from the TFPs’ policy documents. During the formulation phase of the PNPS, there was no knowledge available on cash transfers in Burkina Faso. Yet improving cash transfers was one of the priority strategies of the policy. On the other hand, results of pilot projects for social protection in health were available and helped inform choices. Overall, the strategy formulation essentially amounted to a planning process influenced by the interests of the various ministries and TFPs.

### Issues shaping to the use of explicit knowledge

The ministries tended to favor the use of documents from institutions with which they had developed a long tradition of partnership. The MEF, which drafted the framework memorandum, more often cited World Bank publications. The Ministry of Labour and Social Security used ILO documents to provide conceptual insights into the work of the Social Insurance Commission. Decision-makers showed great interest in knowledge about the economic impact of policies. TFPs were the main intermediaries in making explicit knowledge accessible to national actors through training workshops and government meetings. These moments of interaction fostered exchanges of information and knowledge on social protection, provided an overview of experiences elsewhere, and enabled a consensus on the need for a comprehensive policy in response to the social protection deficit. The information transmitted by the TFPs was most often framed in accordance with their objectives (ignoring information that was contradictory) to support and convey their ideas and opinions. However, decision-makers did not consider information that did not agree with their points of view. In contrast to the World Bank study on social safety nets, which was positively assessed and most often cited by stakeholders as a reference document, the IMF study on the effectiveness of universal hydrocarbon price subsidies was considered invalid and irrelevant to inform decision-making. National stakeholders felt the study had been produced to convince the government of the ineffectiveness of this subsidy and the need for change in favor of IMF ideas. Likewise, the involvement of national actors in the conduct of this study influenced its acceptance. Officials from the MEF and MAS participated and recognized themselves in this study, which was in fact an analytical synthesis of their interventions. “*It really was a project conducted with the main actors working in the domain*, *to achieve this*. *The Bank saw*, *to some extent*, *what actions we had going on in the field and could see why we were implementing them*” [EF-2M]. The document was useful, in that it presented in a coherent and structured way information that was dispersed throughout the reports of the various sectors involved in social protection. The report provided a more overarching and cross-sectoral view of the challenges ahead. This made it possible to transcend the limitations of ministries’ functioning in “silos” and of sectoral government management. The national actors also believed the IMF and the World Bank used research to ensure their opinions and ideas would be taken into account. For those actors, the actions and position of these institutions were grounded in the values they defended. As such, they considered that the IMF’s study necessarily had to support its position by demonstrating the ineffectiveness of subsidies so that government funding would be redirected towards strategies that were more aligned with the IMF’s position: “*How to convince countries to stop the subsidy*? *You need to demonstrate that the subsidy isn’t effective*. *So the study produced results that said these subsidies benefited households that were not poor*” [EF-2M].

The controversy over the results of the IMF study showed that knowledge is unlikely to inform decision-making if the proposed reform goes against the ideas and interests of the ruling political actors. In other words, the non-consonance of certain ideas and knowledge with the political elites’ perceptions of the problem is an obstacle to conceptual or instrumental use. The study recommended the hydrocarbon subsidy be abolished because it was expensive, inefficient, and unfair, benefiting the rich more than others. The study’s results were presented to the Prime Minister (a trained economist), who rejected the recommendation. In fact, the government saw this subsidy not only as a social protection measure, but also as a measure to manage the social consequences of inflation, protests, and social discontent. Implementing the recommendation was seen as likely to increase the social tension that the government was trying to allay. This situation showed a divergence of views between the IMF and the government on the intervention’s objective and beneficiaries. Aside from the knowledge on the effectiveness of the subsidy measure, in rejecting the recommendation the government also took into account the social and political consequences of implementing it. An example cited to justify this position was the social unrest in Nigeria after withdrawal of a similar subsidy: “*In Nigeria they [IMF] managed to convince the government*, *which abandoned it*. *There were riots*, *and a week later they were forced to reinstate it*” [EF-3M].

Both the perceived quality of the studies and the operational relevance of the recommendations fostered use, most often strategic or conceptual. The World Bank report, which was the document most frequently cited in the interviews, was viewed positively. Also, that study’s recommendations were consistent with those proposed in the CSLP assessment: invest in social protection for the poor and vulnerable, and adapt interventions to population needs while taking into account barriers to services use. Social protection was presented as an instrument for poverty reduction and a means of maintaining peace and social cohesion, and thus as a solution to the government’s problem of social tension. Also, doubts about knowledge reliability were an obstacle to instrumental use. The IMF study on hydrocarbon subsidies was not well received. It provoked considerable controversy, and its validity was called into question. INSD officials considered that the measurement tools, not being adapted to the context, were unable to capture the reality of the situation, including how this intervention benefited the poor. The IMF’s method of analysis was considered simplistic: “*For them*, *when we subsidize hydrocarbon prices*, *it’s you and me filling up the fuel tank*, *okay*! *That’s how they read it*. *That’s a very simplistic analysis of the situation*” [EF-MM].

## Discussion

These results are in line with other studies in Africa showing that locally produced knowledge, particularly that resulting from scientific research, has little influence on social health protection policies [63]. Contextual factors, such as financial constraints, lack of trust in local research and political (political) issues negatively influence the use of local research in health policy-making in Africa [63, 64]. Also, this article is an important and enriching contribution to the field of social protection policy studies in Africa. Most of the research in this field has focused on social transfers [9, 10]. To our knowledge, this study is the first of its kind to analyze the development of social protection policies in francophone sub-Saharan Africa other than social protection in health. In addition, the focus on the use of explicit knowledge fills a knowledge gap on how local actors use explicit knowledge to guide the choice of social protection policies and practices.

The results show that national actors have made greater use of internal documents, government reports, and TFP study reports, and have drawn on experiences in Africa and Latin America disseminated by international organizations. Thus, scientific research data published in peer-reviewed journals has been used very little directly. This may be due to the fact that most research on social protection comes from the grey literature commissioned by the TFPs [18, 28, 90].

Instrumental, conceptual, and strategic use of explicit knowledge, mostly from the grey literature, informed the PNPS development. This knowledge informed the two phases of its development, emergence and formulation, in different ways.

Explicit knowledge more prominently informed the emergence phase. National actors deepened and increased their knowledge (conceptual use) on the importance and challenges of social protection. Social protection, a new concept, was presented to local actors as a solution to strengthen social cohesion (a government need). The government had been highly criticized and was confronted with social tensions related in part to the social protection shortfall. The search for a solution to ease social tension and to show solidarity with the most disadvantaged made the government receptive not only to the knowledge available internally, but also to that produced by the TFPs. The World Bank report on social safety nets and the case studies presented during the workshops conveyed knowledge and messages on social protection (reducing inequality and poverty, strengthening social cohesion) that coincided with the aspirations of decision-makers. The political will to resolve the problem heightened the government’s willingness to consider relevant explicit knowledge. Studies and statistical data helped inform local actors on the status of the social protection deficit, which was identified as a public problem requiring government action.

During the formulation phase, the TFPs offered advice and provided technical support when called upon, and funded steering committee meetings and PNPS validation workshops. This reflected the government’s lesser financial and technical autonomy and confirmed the role of international institutions in disseminating ideas and knowledge on social protection [91, 92]. For example, the process of formulating the PNPS 2012 was fully funded by UNICEF [35]. National actors made greater use of ministry activity reports, government reports, and TFP study reports, and drew lessons from the experiences of English-speaking African and Latin American countries disseminated by international organizations. Those organizations targeted and framed explicit knowledge in line with their preferred strategies. In this context, the role of knowledge in the formulation phase was nuanced. Analysis of the formulation process revealed that local actors’ thinking was not guided by whether the solutions had the capacity to solve the problem in the context of Burkina Faso [34]. The choices were not based on an analysis of strategies (effectiveness, equity, unintended effects) and their applicability (cost, acceptability, feasibility). This way of working was due in part to the actors’ limited knowledge on social protection and to the lack of government guidance on strategic choices.

It was also reinforced by the absence of any organizational mechanism to support the drafting committee in a knowledge-informed decision-making process. Such a mechanism, which contributes to knowledge management and strengthens stakeholders’ capacity to integrate knowledge into practices, would have made it possible to go beyond theoretical and conceptual learning to analyze the links between solutions, implementation context, and desired objectives [93–95].

This analysis would have favored a non-mechanical instrumental use of explicit and impartial knowledge, motivated mainly by the improvement of the social protection of populations. Strategic use was clearly identified. Considering instrumental use as, “the product of a synthesis of knowledge or recommendations from experts is directly used in policy development, decision making or in the process of solving a problem” [96]. It consisted in drawing on documents (study reports, evaluations, statistics) to justify the content of the PNPS (setting out the foundations and guiding principles of the PNPS, justifying the need to formulate a policy, illustrating the extent of the social protection deficit and the ineffectiveness of the CSLP, and supporting the feasibility and relevance of a specific strategy).

The results showed that knowledge is unlikely to inform decision-making if the proposed reform goes against the ideas and interests of the ruling political actors. Knowledge is considered in decision-making when there is a consensus on its social and political relevance, as well as its validity, and when the recommendations are convergent with the ideas of policy makers. Studies in Uganda, Malawi, and Zambia have shown that aligning knowledge with the needs and policy priorities of a country’s decision-makers promotes its use [33, 65, 97, 98]. In fact, the development of political solutions to problems often takes the form of explicit competition between the ideas of actors with unequal capacities for influence [99]. Ideas such as causal beliefs about economic, social, and political phenomena [100]. Despite the availability of explicit knowledge on the effectiveness of healthcare fees exemptions, the beliefs and preconceived notions of health professionals and decision-makers have long been barriers to implementing a national free healthcare strategy in Burkina Faso [101]. In Rwanda, community-based health insurance was chosen over other potential solutions to improve social protection in health because it was the option most compatible with the ideas of the actors in power [102]. An analysis of social protection policies in South Africa and Botswana showed that policy makers act on the basis of ideas that are less contentious or that can help them make sense of their situation [103].

## Conclusion

In short, the use of peer-reviewed academic research was not explicitly mentioned in this case in Burkina Faso, in contrast to other types of knowledge, such as national statistical data, reports on government program evaluations, and reports on studies carried out by TFPs (international institutions and NGOs). Through conceptual use, national actors acquired new knowledge, deepened their knowledge and understanding of social protection issues in Burkina Faso, and became convinced of the need to develop a policy. Strategic use involved citing knowledge to justify the utility and feasibility of a PNPS. Instrumental use consisted of drawing from workshop presentations and the contents of study reports to write sections of the PNPS. The consideration of a recommendation based on explicit knowledge was influenced by perceived political gains, i.e., potential social and political consequences.

## Supporting information

S1 Table Chronology of the stages in the development of national social protection policy. (DOCX)

S1 File Interview guides. (DOCX)

## Abbreviations

CSLP: Poverty Reduction Strategic Framework (Cadre stratégique de lutte contre la pauvreté
ILO: International Labour Organization
IMF: International Monetary Fund
INSD: National Institute of Statistics and Demographics (Institut national de la statistique et de la démographie
MAS: Ministry of Social Affairs
MEF: Ministry of the Economy and Finance
NGO: Non-governmental organization
PNPS: National social protection policy (Politique nationale de protection sociale
SPONG: Permanent Secretariat for NGO Coordination (Secrétariat permanent pour la coordination des ONG
TFP: Technical and financial partner
UNICEF: United Nations Children’s Fund
WFP: World Food Programme
