# Supplementary material for: How does explicit knowledge inform policy shaping? The case of Burkina Faso’s national social protection policy
Source: PLoS One. 2023 Apr 27;18(4):e0284950. doi: 10.1371/journal.pone.0284950 (PMC10138829; doi:10.1371/journal.pone.0284950)
Supplement: S1 Table — (DOCX) [file pone.0284950.s001.docx]

| Steps | Period 1 | | | | | Period 2 | | | | | Period 3 | | |
| --- | --- | --- | --- | --- | --- | --- | --- | --- | --- | --- | --- | --- | --- |
|  | 02/10 | 04/10 | 05/10 | 07/10 | 10/10 | 02/11 | 06/11 | 08/11 | 10/11 | 01/12 | 03/12 | 06/12 | 09/12 |
| Establishment of the Interdepartmental Steering Committee (ISC). |  |  |  |  |  |  |  |  |  |  |  |  |  |
| Technical Workshop on Social Protection: Drafting a National Vision |  |  |  |  |  |  |  |  |  |  |  |  |  |
| Development of a roadmap for the Interdepartmental Committee |  |  |  |  |  |  |  |  |  |  |  |  |  |
| 1^e^ CIP session: installation, orientation, operation |  |  |  |  |  |  |  |  |  |  |  |  |  |
| 2^e^ IPC session  Framework note PNPS  Terms of reference for the two specialized commissions (social nets and social insurance)  IPC Rules of Procedure |  |  |  |  |  |  |  |  |  |  |  |  |  |
| Final Report of the Social Insurance Commission |  |  |  |  |  |  |  |  |  |  |  |  |  |
| Final Report of the Commission on Social Safety Nets |  |  |  |  |  |  |  |  |  |  |  |  |  |
| 3^e^ IPC session  Examination of the reports of the specialized commissions  Review of the Draft HPN Plan  Review of the draft PNPS action plan  Review of the timeline for the development of the PNPS |  |  |  |  |  |  |  |  |  |  |  |  |  |
| Working session of the Executive Secretariat [drafting of the PNPS]. |  |  |  |  |  |  |  |  |  |  |  |  |  |
| 4^e^ IPC session: review of the PNPS project |  |  |  |  |  |  |  |  |  |  |  |  |  |
| 5^e^ IPC session:  Review and validation of the PNPS project,  Review of the PNPS Action Plan |  |  |  |  |  |  |  |  |  |  |  |  |  |
| 6^e^ IPC session: validation of the PNPS action plan |  |  |  |  |  |  |  |  |  |  |  |  |  |
| Civil society forum [organized by SPONG]: amendment of the draft PNPS and action plan |  |  |  |  |  |  |  |  |  |  |  |  |  |
| National Forum [organized by MEF]: validation of the PNPS and action plan |  |  |  |  |  |  |  |  |  |  |  |  |  |
| Adoption of the PNPS by the Council of Ministers |  |  |  |  |  |  |  |  |  |  |  |  |  |

**Table I: Chronology of the development stages of the PNPS: February 2010 to September 2012**
