# Supplementary material for: How does explicit knowledge inform policy shaping? The case of Burkina Faso’s national social protection policy
Source: PLoS One. 2023 Apr 27;18(4):e0284950. doi: 10.1371/journal.pone.0284950 (PMC10138829; doi:10.1371/journal.pone.0284950)
Supplement: S1 File — (DOCX) [file pone.0284950.s002.docx]

**PNPS Formulation Interview Guide**

| **Analysis of the formulation of the PNPS= Choice of solutions** |
| --- |

**Respondents:**

- *Government actors who are members of the Inter-Ministerial Committee, members of the Executive Secretariat of the Inter-Ministerial Committee, members of the Commission on Social Nets, members of the Commission on Social Insurance)*
- *Technical and financial partner and UN organization NGO and civil society, local association*

1. **Operation of the Interdepartmental Formulation Committees of the PNPS**

- On the way each level of decision making operates: how was the work of the commissions carried out? How was the content decided and adopted?
- What guidance, direction, or instructions or outline for the writing were given? By whom? And why were they given?

1. **General information on social protection solutions and instruments.**

- Why were the solutions and instruments included in the policies chosen? (Effectiveness, feasibility)
- How do experiences from elsewhere contribute to decision making
- What sources of information were you personally most receptive to?

1. **Development of the content of the PNSP** (solutions, strategies)

- Describe the process of choosing solutions and strategies *for* the HPN *(explore* ***contradictions, points of contention****, and bases for consensus during the drafting process? Which actors have conflicting ideas and why? What are the contradictions within the same group of actors?*

1. **Justification of the choice by instruments and by actors (points of consensus, divergence and arguments of justification)**

- *Why did some of them succeed in getting their idea across and not others? Who are the most influential actors? Why are they influential? And why do they think their solutions are the best?*
- **Factors that influenced the quality of the formulation process.** *Explore stakeholder capacity on social protection, high-level stakeholder participation,*

1. **Exploring the rationale for the selection of the content of the HPN**

**Interview guide on the role of explicit knowledge in the formulation and emergence of the PNSP**

| **Roles of knowledge in emergence and formulation** |
| --- |

*Confirm the interviewee's role in the policy development process (Executive Officer prior to formulation, committee leaders, TFP leader prior to and during formulation).*

1. **Political context**

- Explore how the knowledge (research results, routines data, report ) contributed to shape the perception of the actors on the situation of social protection (knowledge from research and change of perception on the problem)
- Explore how knowledge (research findings, data routines, reports) has contributed to a gradual change in actors' perception of the nature and importance of the social protection issue (people involved, scope, consequences) *(*explain how?)
- Was his knowledge so important or decisive that it prompted actions to be taken to turn it into policy?
- What are the factors of change (identify the events, changes, situation) that contributed to the use or not of knowledge and the consideration of knowledge in the formulation of the PNPS?
- *Situations in the context that resulted in a need for knowledge*
- *Putting the problem on the agenda and providing a solution.*
- *Climate of public opinion and change of political current (a new vision, speeches and ideas on the PS).*

1. **Actors and the use of knowledge**

- Who were the main people and groups of people, whose actions led to the formulation of the PNPS.
- Was knowledge used at any point in the process *(by whom? What types of knowledge, when it was used, why it was used)*
- Explore times and frameworks for knowledge sharing by actors (government actors, TFPs and civil society? to access knowledge): workshop, training, meeting, study tour, etc.)
  - *Was there any training or sharing of information or knowledge about social protection before and during the writing? Who participated? Who provided the training or shared their knowledge? Did the training change your view of social protection (in what way?)? How did this training contribute to the PNSP?*
- How did participation in training workshops, technical workshops, provide access to research knowledge (paper received, presentation attended)?
- How did the training workshops, the technical workshops influence the knowledge of the actors on social protection?
- How did the training workshops, the technical workshops influence the writing of the content of the PNPS?
- How was the knowledge used (problem definition, choice of solutions/formulation?)

1. **Explicit knowledge**

- How did explicit knowledge influence the development of the HPNP (use previous interviews, explore reasons if response suggests research played a role or not).
- The emergence of the PNPS (knowledge about the social protection problem)
- The formulation of the PNPS (drafting of the policy content?)
- What types of knowledge contributed to the development of the PNPS (emergence, formulation)
- After the initial response, ask about any of the following: national or international scientific literature; research reports; briefs of research results obtained by researchers; literature syntheses; direct communication with individual researchers; participation in seminars where research results were presented; liaison with research centers; briefs of policy advisors or government officials; pilot project reports, evaluation reports, working papers of international organizations.
- Explore types of uses (ask for examples of instrumental, conceptual, and strategic types of uses)
- What types of knowledge were most used? How and under what circumstances?
- Are there reasons why knowledge does not influence policy to the extent that it might have? (Limitations to knowledge use)
- Competence and ability of decision-makers to use explicit knowledge (level of training, their knowledge of research methods ;
- How do you perceive the roles of international actors and organizations? (Explore the role of knowledge transfer, pressure for instrument choices)
